# Cryo-EM structure of the calcium-sensing receptor complexed with the kokumi substance γ-glutamyl-valyl-glycine

**DOI:** 10.1038/s41598-025-87999-1

**Published:** 2025-01-31

**Authors:** Hiroki Yamaguchi, Seiji Kitajima, Hiroshi Suzuki, Shota Suzuki, Kouki Nishikawa, Akiko Kamegawa, Yoshinori Fujiyoshi, Kazutoshi Takahashi, Uno Tagami, Yutaka Maruyama, Motonaka Kuroda, Masayuki Sugiki

**Affiliations:** 1 Ajinomoto Co., Inc., 1-1 Suzuki-cho, Kawasaki-ku, Kawasaki, Kanagawa 210-8681 Japan; 2 Advanced Research Initiative, Institute of Integrated Research, Institute of Science Tokyo, 1-5-45 Yushima, Bunkyo-ku, Tokyo, 113-8501 Japan; 3 CeSPIA Inc., 2-1-1 Otemachi, Chiyoda-ku, Tokyo, 100-0004 Japan; 4 Joint Research Course for Advanced Biomolecular Characterization, Faculty of Agriculture, Tokyo University of Agriculture and Technology, 3-5-8 Saiwai-cho, Fuchu, Tokyo 183-8509 Japan

**Keywords:** Calcium-sensing receptor, γ-Glutamyl-valyl-glycine, Class C GPCR, Cryo-electron microscopy, Single particle analysis, *Koku* perception, Electron microscopy, Biochemistry

## Abstract

Taste is a key element for food palatability and is strongly influenced by the five basic tastes and other taste sensations, such as fatty orosensation, and koku perception, which indicates taste complexity, mouthfulness and lastingness. This study focuses on the taste modifier γ-glutamyl-valyl-glycine (γ-EVG), a potent kokumi substance that enhances taste and koku perception by modulating the calcium-sensing receptor (CaSR). We used cryo-electron microscopy to determine the structure of the CaSR/γ-EVG complex at a resolution of 3.55 Å. Structural analysis revealed important interactions between γ-EVG and the CaSR, involving key residues, such as Pro39, Phe42, Arg66, Ser147, and Glu297. Mutagenesis experiments demonstrated the importance of these residues in peptide binding. Each γ-EVG residue contributed to its binding to the orthosteric ligand binding site of the CaSR. These findings elucidate the molecular basis of kokumi peptide recognition by the CaSR and contribute to a better understanding of positive allosteric modulators of the CaSR. In addition, this research provides valuable insights into the functionality of class C G-protein-coupled receptors in taste perception, potentially informing the development of new taste modifiers and advancing the field of food science.

**Supplementary Information:**

The online version contains supplementary material available at 10.1038/s41598-025-87999-1.

## Introduction

The human sense of taste has a huge effect on the palatability of food. The five basic tastes are the main factors responsible for the perception of taste^1^, and they are perceived through the activation of taste receptors expressed in taste cells on the tongue^1,2^. However, describing the taste of foods in terms of these five basic tastes is too simplistic. Koku perception is often used in Japan to describe a positive sensation that differs from the basic tastes and contributes to palatability^3^. The scientific definition of koku perception, proposed by the Nishimura group, refers to the combined and well-balanced sensation perceived when the senses of taste, smell, and texture are more intensely stimulated^4^. They also defined the three basic attributes of koku perception, as complexity, mouthfulness, and lastingness and have proposed that foods with any of these three sensory attributes are described as having koku perception^3–5^. Kokumi substances are taste-related koku-perception-enhancing substances, such as γ-glutamyl peptides like γ-glutamyl-valyl-glycine (γ-EVG). They are taste modifiers that do not have a taste themselves, but when mixed with taste substances, they enhance the complexity, mouthfulness, and lastingness^5–16^. We have previously reported that the calcium-sensing receptor (CaSR), which is expressed in taste cells^17,18^, is involved in the perception of kokumi substances^5,11,18^. CaSR is a seven-transmembrane G-protein-coupled receptor (GPCR) and belongs to the same class C subfamily that includes taste receptors T1R1 and T1R3^2,19,20^. CaSR is a receptor that has been shown to be expressed in a variety of animal species^21^ and humans, and is expressed in several types of tissue, including parathyroid gland and kidney tissue. CaSR was originally identified as being involved in regulating plasma calcium levels and is central in calcium homeostasis^22^. CaSR functions as an obligate homodimer and has a Venus flytrap (VFT) domain, a bilobed structure responsible for ligand binding, and an N-terminal extracellular domain linked to the seven-transmembrane domain by a cysteine-rich domain^23–25^. CaSR is activated not only by calcium ions but also by a wide range of amino acids and γ-glutamyl kokumi peptides^6,11,12,26–28^. We have recently found that γ-EVG functions as a positive allosteric modulator (PAM) for CaSR, similar to some amino acids^6,29,30^. CaSR has various ligand-binding sites, and various amino acids can bind to and activate the VFT domain of CaSR^23–25^. Structures of CaSR complexed with L-tryptophan and its derivatives have been determined by X-ray crystallography and cryo-electron microscopy (cryo-EM), and the structures have revealed the interaction with the VFT domain and the conformational changes in the domain induced by ligand binding^31–33^. Some molecular docking analyses have also been reported^34^. However, the structural properties and recognition mechanisms for peptides, especially γ-glutamyl kokumi peptides that function as potent taste modifiers, have remained unclear.

Here, we report a structure of the CaSR complexed with γ-EVG experimentally determined by cryo-EM at an overall resolution of 3.97 Å^35^. We obtained clear density maps at a resolution of 3.55 Å by local refinement covering only the VFT-domain-bound γ-EVG. The structural information agreed well with our peptide screening results from previous studies^11,36^. Furthermore, the structure–activity relationship of CaSR was discussed in detail based on CaSR mutant assays. These results will not only clarify how the CaSR recognizes kokumi peptides but may also provide important fundamental information about PAMs that interact with the VFT domain of the CaSR.

## Results and discussion

### Structure of CaSR with γ-EVG

**Fig. 1:**
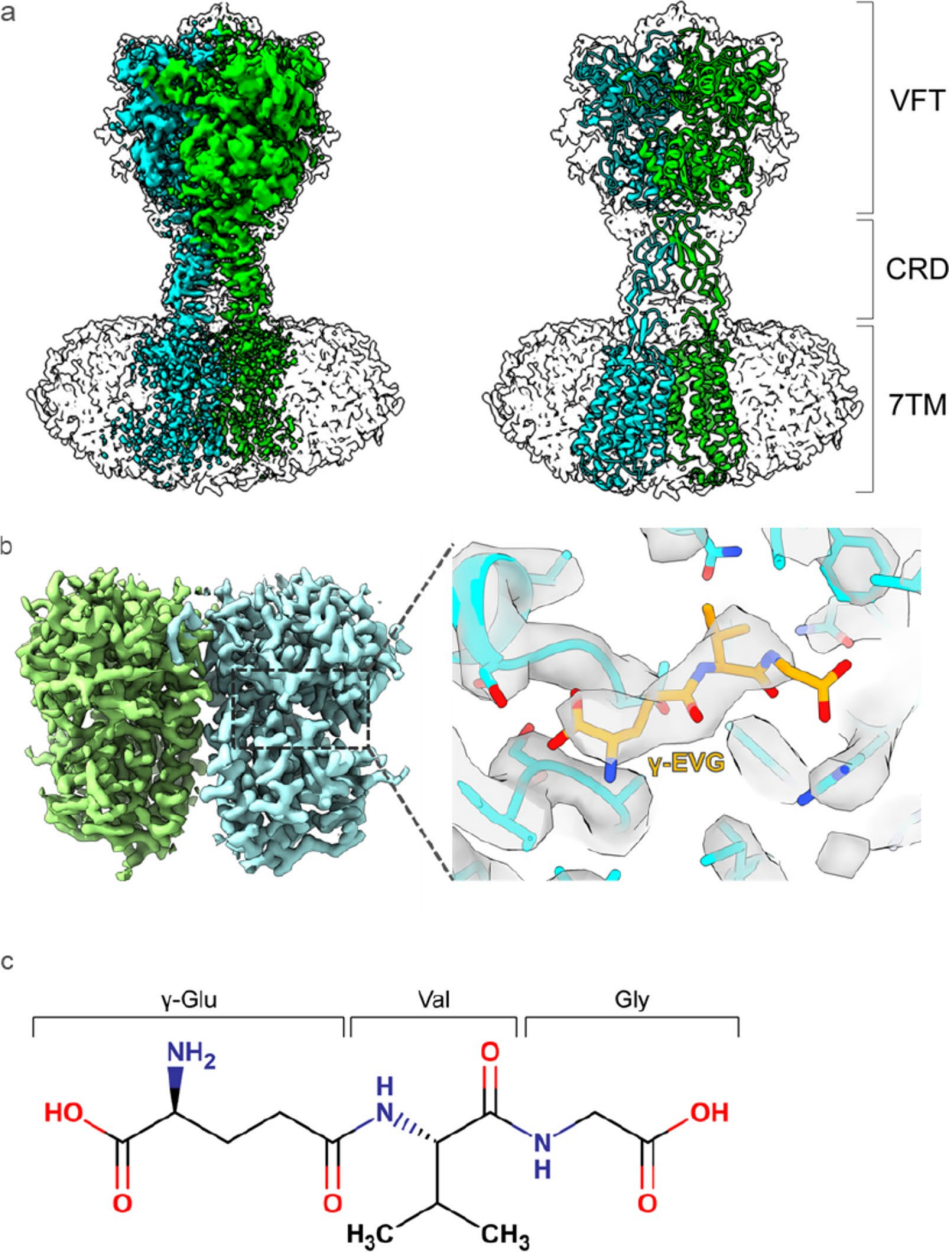
Cryo-EM map of the CaSR/γ-EVG complex. (**a**) Overall density map of CaSR (left) and cartoon of the model of CaSR in an active form (PDB: 7m3G) (right). CaSR is composed of a VFT domain, a CRD, and a 7TM domain. (**b**) Focus-refined map of the VFT domain of CaSR. Stick model of γ-EVG (orange) is overlaid with the cryo-EM density. (**c**) Chemical structure of γ-EVG. *Cryo-EM* cryo-electron microscopy, *CaSR* calcium-sensing receptor, *γ-EVG* γ-glutamyl-valyl-glycine, *VFT* Venus flytrap, *CRD* cysteine-rich domain, *7TM* seven-transmembrane.

CaSR was expressed in Sf9 insect cells and purified with an affinity tag and by subsequent size-exclusion chromatography. By adding calcium ions, γ-EVG, and two PAMs, etelcalcetide and evocalcet^31^, we obtained a cryo-EM map of the active state of CaSR at a resolution of 3.97 Å (Fig. 1a). To obtain more detailed structural information about the binding mode of γ-EVG binding to CaSR, local refinement of the VFT domain was performed, and the density map resolution was improved to 3.55 Å (Fig. 1b, c and Supplementary Fig. S1). The ligand density of γ-EVG was identified in the amino acid binding pocket of CaSR (Fig. 1b). Etelcalcetide binds covalently to the Cys482 residue^31^. However, in our map, the cryo-EM density corresponding to etelcalcetide was weak, and an accurate model of etelcalcetide could not be built (Supplementary Fig. S2). In the following discussion, we use the complex structure of the VFT domain obtained by local refinement.

### Interactions of γ-EVG

**Fig. 2:**
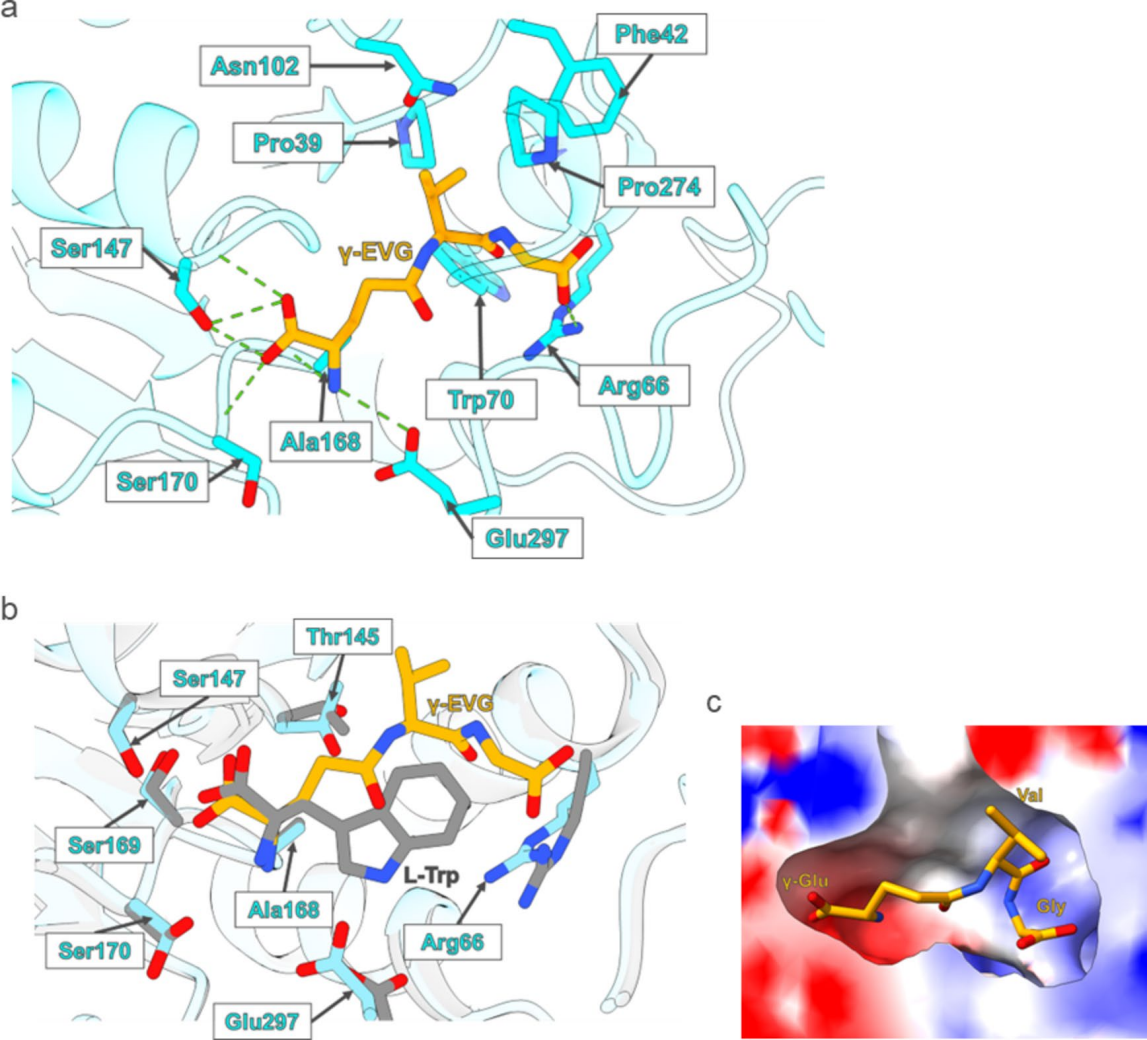
γ-EVG recognition CaSR. (**a**) Interactions between CaSR and γ-EVG. Hydrogen bonds are shown as green dotted lines using ChimeraX^46^. CaSR is shown as a cartoon model (cyan) and the side chains of residues that interact with γ-EVG are shown as stick models (orange). (**b**) Superimposed models of CaSR/γ-EVG (cyan) and CaSR/L-Trp (PDB: 7M3G) (gray) based on the structure of CaSR. Residues within 4 Å of L-Trp are labeled and shown as stick models. γ-EVG and L-Trp are also shown as stick models (orange and gray). (**c**) Surface model of CaSR around γ-EVG (orange). The electrostatic potential is indicated by color (red for negative potential through white to blue for positive potential). *CaSR* calcium-sensing receptor, *γ-EVG* γ-glutamyl-valyl-glycine.

In previous work, screening of dipeptides and tripeptides based on the CaSR activation potency identified γ-EVG as having the most potent activity among those tested^11,36^, and the amino and carboxyl groups at the γ-Glu position were found to be important for recognition by CaSR. In our cryo-EM structure, the amino group of γ-Glu, namely the N-terminus of γ-EVG, formed a hydrogen bond with the main chain of Ala168 and the side chain of Glu297. The carboxyl group of γ-Glu formed a hydrogen bond with the side chain of Ser147 and the main chains of Ser147 and Ser170 (Fig. 2a). Comparison with the reported structure of CaSR complexed with L-Trp (PDB: 7M3G)^32^ showed that the root mean square deviation of Cα atoms between two VFT domains was 0.559 Å. The positions of the amino group in the free amino acid L-Trp and the carboxyl group in the γ-Glu residue in γ-EVG were similar (Fig. 2b). The amino group of L-Trp formed a hydrogen bond with Ala168 and Ser170, and the carboxyl group formed a bond with the Ser147 and Ser170 (Supplementary Fig. S3). However, the binding site of the L-Trp side chain was different from that of γ-EVG, and among the neighboring residues, there were slight differences in the position of the Arg66 and Glu297 side chains (Fig. 2b). Glu297 of CaSR had no hydrogen bond with L-Trp. These results suggested that the hydrogen-bond interactions of γ-Glu with three residues (Ala168, Ser147, and Ser170) were conserved and may be important in the binding of amino acids and γ-Glu peptides to CaSR.

The valine side chain of γ-EVG was 3.9, 4.3, 5.2, and 4.5 Å away from the Pro39, Phe42, Trp70, and Pro274 side chains, respectively (Fig. 2a and Supplementary Fig. S4), indicating that these hydrophobic residues formed a hydrophobic cluster and may be involved in the interaction of γ-EVG with valine. Previous studies have shown that changing the valine of γ-EVG to a basic, acidic, or large hydrophobic residue resulted in loss of activity, whereas replacing valine with hydrophobic and small hydrophilic side chains retained the activity for CaSR^11,36^. Only valine and norvaline at position 2 in γ-EVG maintained high levels of peptide activity at the CaSR, suggested that the second residue of the tripeptide may be tightly bound to the pocket by forming hydrophobic interactions with the hydrophobic cluster of CaSR.

The carboxyl group of the glycine of γ-EVG formed a hydrogen bond with the Arg66 side chain. It has been reported that the presence of a carboxyl group in the glycine position increased activity markedly, whereas the side chain was less important because many amino acids, except Ile, Tyr, Trp, and β-Ala, were tolerated in the glycine position^36^. The critical role of Arg66 in the recognition of the carboxyl group of the tripeptide in our CaSR/γ-EVG complex structure was consistent with the structure–function relationship shown in previous studies^36^. Additionally, the charge around γ-EVG showed that the binding region of glycine was positively charged, supporting the importance of charge for carboxyl group binding (Fig. 2c). In contrast, the binding site of γ-Glu was negatively charged, suggesting that the formation of hydrogen bonds with the amino and the carboxyl groups was the important factor, not the charge of the compound.

### Mutation of the binding pocket residues

To investigate the contribution of each residue at the binding site to the activity of γ-EVG, the interacting residues Arg66, Ser147, and Glu297 were mutated to alanine. The charge of Arg66 appeared to be important for the interaction (Fig. 2a); therefore, Arg66 was also mutated to a negatively charged glutamate residue. The hydrophobic residues around the valine side chain in γ-EVG, Pro39 and Phe42, which can form hydrophobic interactions with valine, were mutated to a hydrophilic residue, serine (Fig. 2a). We also focused on Asn102, which was 4.0 Å from the same tertiary carbon. Previous studies have suggested that Asn102 may be involved in the interaction with peptides^34^. Here, we investigated the mutational effect of replacing Asn102 with a hydrophobic bulky amino acid on substrate binding.

The expression levels of each mutant were analyzed in a surface expression assay (Supplementary Fig. S5). The expression level of the S147A mutant was substantially lower than the wild type, suggesting that this mutation affected the folding and/or stability of the CaSR. The expression levels of P39S and N102F were reduced to 59.6% and 65.2% of that of the wild type, respectively, whereas the expression levels of the E297A, F42S, R66A, and R66E mutants were more than 80% of the wild-type expression. Therefore, in the following activity assays, we excluded the S147A mutant, the fluorescence intensity of which was similar to that of the negative control (mock).

**Fig. 3:**
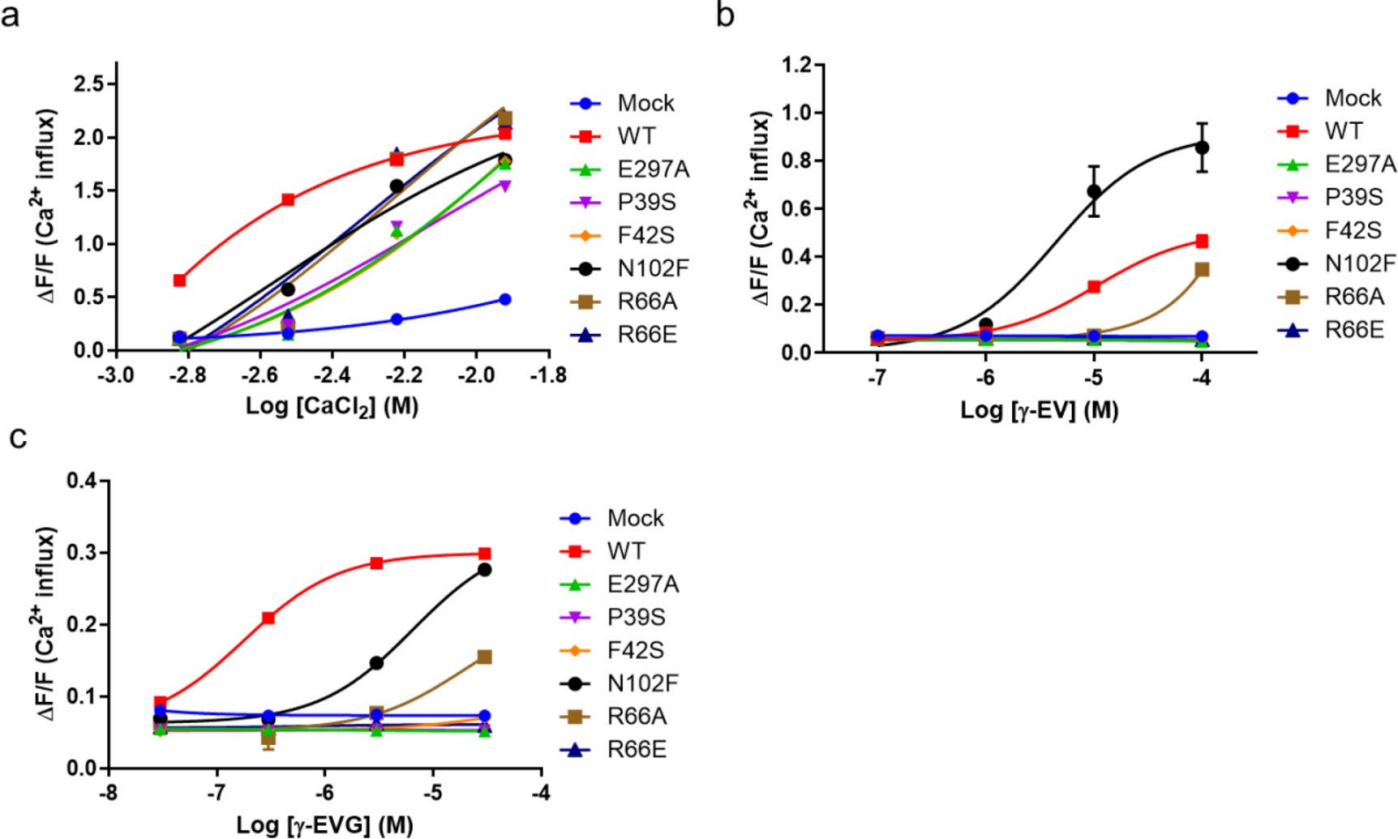
Dose-response curves of key residue mutations in ligand binding pockets and receptor activation for peptides in CaSR in a calcium mobilization assay. (**a–c**) Dose–response curves of key residue mutations in CaSR in response to stimulation with CaCl_2_ and g-glutamyl peptides in a calcium mobilization assay. Data are mean ± s.e.m. from three independent experiments (*n* = 3). (**a**) CaCl_2_, (**b**) γ-EV, and (**c**) γ-EVG. *CaSR* calcium-sensing receptor, *γ-EV* γ-glutamyl-valine,*γ-EVG* γ-glutamyl-valyl-glycine, *WT* wild type.

**Fig. 4:**
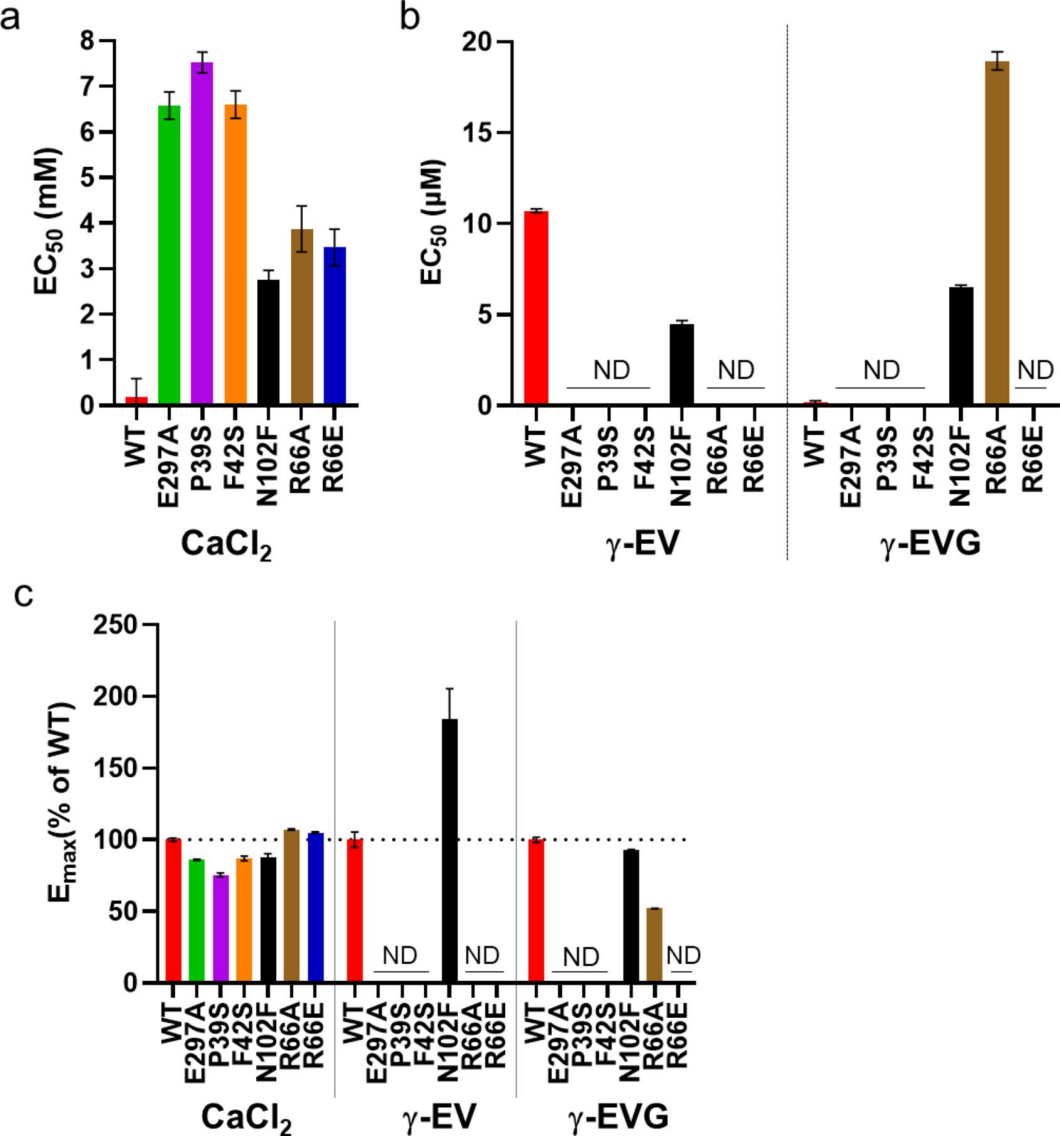
Ca^2+^ mobilization assay responses in wild-type CaSR and mutants. (**a**,** b**) EC_50_ values were calculated from the concentration–response curves (Fig. 3 and Supplementary Fig. S6). Data are means from three independent experiments (*n* = 3). (**c**) *E*_max_ values (% of wild-type values) were calculated from the concentration–response curves (Fig. 3 and Supplementary Fig. S6). Data are shown as mean ± s.e.m. from three independent experiments (*n* = 3). The horizonal dotted line shows *E*_max_ of the wild type. *CaSR* calcium-sensing receptor, *E*_*max*_ maximum response, *γ-EV* γ-glutamyl-valine, *γ-EVG* γ-glutamyl-valyl-glycine, *WT* wild type, *ND* not detected.

We examined the responses of PEAK^rapid^ cells expressing the six mutants against various CaSR ligands (Fig. 3 and Supplementary Fig. S6). In response to CaCl_2_ stimulation, all mutants had an EC_50_ that was one order of magnitude lower than that of the wild type (Fig. 4a and Supplementary Table S1). The six mutants showed weaker CaCl_2_ binding activity compared with the wild type (Fig. 4c and Supplementary Table S2). The results suggest that the generated mutants are responsive to calcium and function as CaSR receptor, although their responsiveness is attenuated. Next, L-Tyr, γ-EV and γ-EVG were evaluated. L-Tyr is evaluated as an amino acid, but L-Trp was excluded from the evaluation due to the low solubility in the assay buffer. In the wild type, the EC_50_ value for γ-EVG was the lowest at 0.18 ± 0.1 µM, and a weak response was observed for L-Tyr and γ-EV (Figs. 3 and 4b, Supplementary Fig. S6 and Table S1). We have previously reported similar EC_50_ values for these ligands^11^. These ligands have been reported to act as PAMs against CaSR^6,11^. These higher activities than those reported by Laffitte et al. appeared to be due to the calcium mobilization assay being performed at a twofold higher calcium concentration than theirs^21^. There was no response to L-Tyr or γ-EV for the mutants, except N102F. The N102F mutant was more active than the wild type with L-Tyr and γ-EV (Fig. 3 and Supplementary Fig. S6). The reason for this result is unknown, but the mutation to the Phe residue, which is bulkier than Asn, may fill the vacancies in the valine and γ-Gly binding sites of γ-EVG, resulting in stronger binding of the amino acid and dipeptide.

The results for γ-EVG were different from those for the other ligands. The E297A mutant, which interacted with γ-Glu, greatly reduced the response and its EC_50_ could not be calculated. In addition, the mutations of Pro39 and Phe42 to serine caused a large decrease in the response of Pro39, and the EC_50_ could not be calculated (Fig. 4b). Both residues could form hydrophobic interactions with valine in γ-EVG, and the distance to valine from Phe42 was larger than that from Pro39; thus, the effect on the interaction with γ-EVG was expected to be smaller than that of the P39S mutation (Fig. 4b, c). The EC_50_ of the N102F mutant was 6.52 ± 0.1 mM for γ-EVG, approximately 36.2-fold higher (6.52 / 0.18 = 36.22) than the wild-type EC_50_ (0.18 ± 0.1) (Fig. 3 and Supplementary Table S1). Unlike L-Tyr and γ-EV, the N102F mutant did not show lower EC_50_ values for γ-EVG than the wild type (Fig. 4b and Supplementary Fig. S6). This result suggests that hydrophobic interactions of valine with Pro39 and Phe42 contributed more than those with N102 in γ-EVG. For Arg66, which interacted with Gly in γ-EVG, mutation to alanine reduced its function compared to the wild-type, but mutation to a negatively charged glutamate residue completely abolished the activity (Fig. 4b, c and Supplementary Table S1). This observation strongly suggests that both the hydrogen bond formation with Arg66 and the positive charge of Arg66 affect the γ-EVG binding. Of the residues proposed in the previous study to bind γ-EVG to the CaSR, N102 and E297 were reported by Laffitte et al. to have the same amino acid sequence in cat and human and may be one of the key residues in the CaSR activation in both species^21^. Therefore, the present study validates their speculation and the finding of residues contributing to CaSR activation in kokumi substances may help in the discovery of more potent CaSR-activating kokumi substances.

In conclusion, our results provide new insights into the role of each residue in the function of amino acids and peptides as PAMs for CaSR. These findings may provide important fundamental information about the functional structure of class C GPCRs. It may also contribute to the design of more potent kokumi substances and the development of seasonings and foods with koku perception containing kokumi substances.

## Materials and methods

### Protein expression and purification

Human CaSR (Uniprot: P41180, residues 20–894) with N-terminal influenza haemagglutinin signal peptide followed by FLAG-tag (DYKDDDDK) and a three-Ala linker was expressed from pFastBac1 vector (Themo Fisher Scientific, Waltham, MA, USA) as previously described^32^. The CaSR was expressed in Sf9 insect cells using the Bac-to-Bac Baculovirus Expression System (Themo Fisher Scientific). Sf9 cells grown at a density of 2 × 10^6^ to 3 × 10^6^ cells/mL in Sf-900 III SFM medium (Themo Fisher Scientific) were infected with the baculovirus-expressed CaSR. Forty-eight hours after infection, cells were harvested and washed once with HEPES-buffered saline (HBS; 20 mM HEPES 7.5, 150 mM NaCl). The pellets were stored at − 80 °C. Cell pellets were lysed by homogenization in HBS with 10 mM CaCl_2_, 1 mM γ-EVG, protease inhibitors (Roche, Basel, Switzerland), DNase I, 12.5 µM etelcalcetide, and 20 µM evocalcet. The membranes were solubilized by the addition of 1% *n*-dodecyl-β-maltoside (DDM; Anatrace, Maumee, OH, USA) and 0.2% cholesteryl hemisuccinate Tris salt (CHS; Anatrace) for 1 h at 4 °C. The supernatant was cleared by centrifugation and incubated with M2 FLAG resin (Merck, Darmstadt, Germany) for 1 h. After binding, the resin was washed with 10 column volumes of HBS containing 10 mM CaCl_2_, 1 mM γ-EVG, 12.5 µM etelcalcetide, 20 µM evocalcet, 0.1% DDM, and 0.02% CHS. The detergent was gradually exchanged to 0.1% glyco-diosgenin (GDN; Anatrace) and 0.01% CHS. The detergent concentration was then lowered to 0.005% GDN and 0.0005% CHS, and Flag-tagged CaSR was eluted from the column with the same buffer containing 0.2 mg/mL Flag peptide (Merck, Darmstadt, Germany). The complex was purified by size-exclusion chromatography on a Superose 6 10/300 column (Cytiva, Marlborough, MA, USA) in HBS with 10 mM CaCl_2_, 1 mM γ-EVG, 12.5 µM etelcalcetide, 20 µM evocalcet, 0.005% GDN, and 0.0005% CHS. Peak fractions were concentrated. γ-EVG was added to the concentrated CaSR to a concentration of 9 mM. The final concentration of CaSR was about 7.8 mg/mL.

### Data acquisition and image processing

Glow-discharged Quantifoil holey carbon grids (R1.2/1.3, Au, 300 mesh) were loaded with 7.8 mg/mL proteins (3.5 µL), and the loaded grids were blotted for 3.0 s at 4 °C and plunge-frozen in liquid ethane using a vitrification system (Vitrobot Mark IV, Thermo Fisher). Data collection was performed on an electron microscope (JEM-Z320FHC, JEOL, Tokyo, Japan) equipped with a cold field-emission gun, an in-column energy filter, and a specimen stage cooled by liquid nitrogen at an acceleration voltage of 300 kV. All images were recorded on a direct electron detector camera (K2 Summit, Gatan, Pleasanton, CA, USA) operated in counted-resolution mode. SerialEM was used for automatic data acquisition^37^. The pixel size was 1.005 Å at the specimen level. The dose rate was limited to 7.98 e-/Å^2^/s at the specimen level. The exposure time was 8.0 s and was subdivided into 40 frames without a pre-dose delay, resulting in an accumulated dose of 63.8 e-/Å^2^. The two datasets were processed using RELION-4.0^38^, and total 6339 image stacks were subjected to beam-induced motion correction using Warp^39^. Contrast transfer function (CTF) parameters for each micrograph were estimated using CTFFIND4^40^. The auto-picked particles from the first dataset were subjected to two-dimensional (2D) classifications, and representative class averages with fine structural detail were selected for the template-based auto picking. The initial 877,546 and 769,428 particles were extracted from the first and second datasets, respectively, with downsampling to a pixel size of 3.015 Å and subjected to 2D classifications. For each dataset, several cycles of three-dimensional (3D) classification were performed with C1 symmetry, and then a total of 177,479 particles were merged and re-extracted to a pixel size of 1.005 Å. The map of the CaSR active state (EMD-23654) was used as a reference map. Because subsequent 3D auto-refinement with C1 symmetry yielded a map with a twofold symmetry, the particles were subjected to 3D auto-refinement with C2 symmetry to improve the map resolutions. Following CTF refinement and Bayesian polishing, a final round of 3D auto-refinement (C2 symmetry) and post-processing yielded the map at an overall resolution of 3.97 Å according to the Fourier shell correlation (FSC) = 0.143 criterion^35^. For local refinement of the VFT domain, a mask for the VFT domain was generated and low-pass filtered to 15 Å. Using this mask, 3D classification was performed with C2 symmetry. The particles were also subjected to 3D auto-refinement with C2 symmetry and a final round of 3D auto-refinement (C2 symmetry) and post-processing yielded the map at an overall resolution of 3.55 Å according to the FSC = 0.143 criterion^35^.

### Model building and refinement

Model building using Coot^41^ and real-space refinement in PHENIX^42^ were iterated for several cycles. Refinement in REFMAC5^43^ in the Servalcat^44^ pipeline was then performed. The final model was inspected visually for its general fit to the map, and the geometry was evaluated further using MolProbity^45^. The final refinement statistics are summarized in Table 1. The molecular graphics figures were prepared using UCSF ChimeraX^46^, UCSF Chimera^47^, and PyMOL version 3.0.2 (Schrödinger, Inc., New York, NY, USA).

**Table 1:** Cryo-EM data collection, refinement, and validation statistics.

	CaSR/γ-EVG complex (EMDB: EMD-61204) (PDB: 9J7I)
Microscope	JEM-Z320FHC
Detector	K2 summit
Magnification	50,000
Voltage (kV)	300
Exposure time (s)	8.0
Electron exposure (e^−^/Å^2^)	63.2
Defocus range (µm)	− 1.0 to − 2.0
Pixel size (Å)	1.005
Symmetry imposed	C2
Initial particle images (no.)	3,387,866
Final particle images (no.)	177,479
Map resolution (Å)	3.55
FSC threshold	0.143
Refinement	
Model composition in the asymmetric unit	
Protein residues	484
Protein atoms	3933
γ-EVG	1
R.m.s. deviations	
Bond lengths (Å)	0.010
Bond angles (°)	1.433
Validation	
Molprobity score	1.76
Clashscore	8.03
Poor rotamers (%)	0.74
Ramachandran plot	
Favored (%)	95.4
Allowed (%)	4.6
Disallowed (%)	0

*Cryo-EM* cryo-electron microscopy, *CaSR* calcium-sensing receptor, *γ-EVG* γ-glutamyl-valyl-glycine, *FSC* Fourier shell correlation, *r.m.s* root mean square.

### ELISA cell-surface expression assay for CaSR

The cell-surface expression of CaSR and its mutants were measured by ELISA chemiluminescence. Cells 48 h post-transfection were plated in poly-D-lysine-coated white 96-well plates (152037, Corning, Corning, NY, USA) and were fixed with 10% (v/v) formaldehyde (50 µL per well) for 10 min at room temperature. The cells were washed twice with phosphate-buffered saline (PBS; 100 µL per well) and incubated with 5% (v/v) bovine serum albumin (BSA) in PBS (100 µL per well) for 30 min. Cells were incubated with an anti-Flag–horseradish peroxidase-conjugated antibody (A8592, Merck) diluted 1:10,000 in 5% (v/v) BSA in PBS for 1 h at room temperature. After washing three times with PBS (100 µL per well), Super Signal Enzyme-Linked Immunosorbent Assay Pico Substrate (50 µL per well, 37070, Thermo Fisher Scientific) was added to each well to develop the signal and the luminescence was counted using a multi-plate reader (i3x SpectraMax, Molecular Devices, San Jose, CA, USA). The luminescence signal was analyzed in GraphPad Prism 6.07 and data were normalized to the wild-type CaSR signal.

### Calcium mobilization assay

The CaSR receptor responsiveness was assessed by using PEAK^rapid^ cells (ATCC, Manassas, VA, USA) transiently transfected with the receptor DNA by the method reported by Kitajima et al.^6^. Briefly, human CaSR or its mutant cDNA with a signal sequence (MAFYSCCWVLLALTWHTSA) followed by FLAG-tag (DYKDDDDK) were inserted into pcDNA 3.1 expression vectors in Opti-MEM I medium (Thermo Fisher Scientific), mixed with FuGENE 6 (Roche), and poured onto PEAK^rapid^ cells grown at a submaximal concentration. After culturing for 24 h in 96-well plates, the cells were incubated with 5 µM Calcium-5 solution (Calcium-5 assay kit, Molecular Devices) for 45–60 min, and measurements were performed using a plate reader (FDSS/µCELL, Hamamatsu Photonics, Shizuoka, Japan) and its associated software. The activation of CaSR expressed in cells increases the intracellular Ca^2+^ concentration, which is measured using a Ca^2+^-sensitive dye. The binding of Ca^2+^ to the Ca^2+^-sensitive dye increases dye fluorescence, with excitation at 485 nm and emission at 525 nm. Test compounds were dissolved and administered in the assay buffer (146 mM NaCl, 1 mM MgSO_4_, 20 mM HEPES, 1 mM CaCl_2_, 2.5 mM probenecid, and 1.39 mM glucose, pH 7.4). Transiently transfected cells were challenged with compounds and compared with the vehicle alone and/or mock-transfected cells. The concentration dependence of the fluorescence intensity (influx of Ca^2+^) was analyzed using the plate reader at 1 s intervals for 180 s. For the dose–response curve calculations, peak fluorescence responses after the addition of compounds were corrected for, normalized to the background fluorescence (Δ*F*/*F* = –*F* − *F*_0_)/*F*_0_), and baseline noise was subtracted. Dose–response curves were calculated by non-linear regression using GraphPad PRISM 8.0.

## Electronic supplementary material

Below is the link to the electronic supplementary material.

Supplementary Material 1

## Data Availability

The map and model have been deposited in the Protein Data Bank (accession number: 9J7I) and the Electron Microscopy Data Bank (accession number: EMD-61204).
