# Supplementary material for: Cryo-EM structure of the calcium-sensing receptor complexed with the kokumi substance γ-glutamyl-valyl-glycine
Source: Sci Rep. 2025 Jan 31;15:3894. doi: 10.1038/s41598-025-87999-1 (PMC11785791; doi:10.1038/s41598-025-87999-1)
Supplement: Supplementary file 1 — Supplementary Material 1 [file 41598_2025_87999_MOESM1_ESM.docx]

Cryo-EM structure of the calcium-sensing receptor complexed with the kokumi substance γ-glutamyl-valyl-glycine

Hiroki Yamaguchi^1,2,§,*^, Seiji Kitajima^1,§,**^, Hiroshi Suzuki^2^, Shota Suzuki^2^, Kouki Nishikawa^3,4^, Akiko Kamegawa^2,3,4^, Yoshinori Fujiyoshi^2,3,4^, Kazutoshi Takahashi^1^, Uno Tagami^1^, Yutaka Maruyama^1^, Motonaka Kuroda^1^ & Masayuki Sugiki^1^

^1^Ajinomoto Co., Inc. 1-1 Suzuki-cho, Kawasaki-ku, Kawasaki 210-8681, Japan

^2^Advanced Research Institute, Tokyo Medical and Dental University, 1-5-45 Yushima, Bunkyo-ku, Tokyo 113-8501, Japan

^3^CeSPIA Inc., 2-1-1 Otemachi, Chiyoda-ku, Tokyo 100-0004, Japan

^4^Joint Research Course for Advanced Biomolecular Characterization, Faculty of Agriculture, Tokyo University of Agriculture and Technology, 3-5-8 Saiwai-cho, Fuchu, Tokyo 183-8509, Japan

^§^These authors contributed equally

Corresponding authors

^*^Hiroki Yamaguchi

Ajinomoto Co., Inc, Kawasaki, Kanagawa 210-8681, Japan

Email: hiroki.yamaguchi.wk7@asv.ajinomoto.com

Tel: +81-70-1054-7875

Fax: +81-44-210-5872

^**^Seiji Kitajima

Ajinomoto Co., Inc, Kawasaki, Kanagawa 210-8681, Japan

Email: seiji.kitajima.in4@asv.ajinomoto.com

Tel: +81-80-2160-4915

Fax: +81-44-246-6196

**Supplementary information**

**
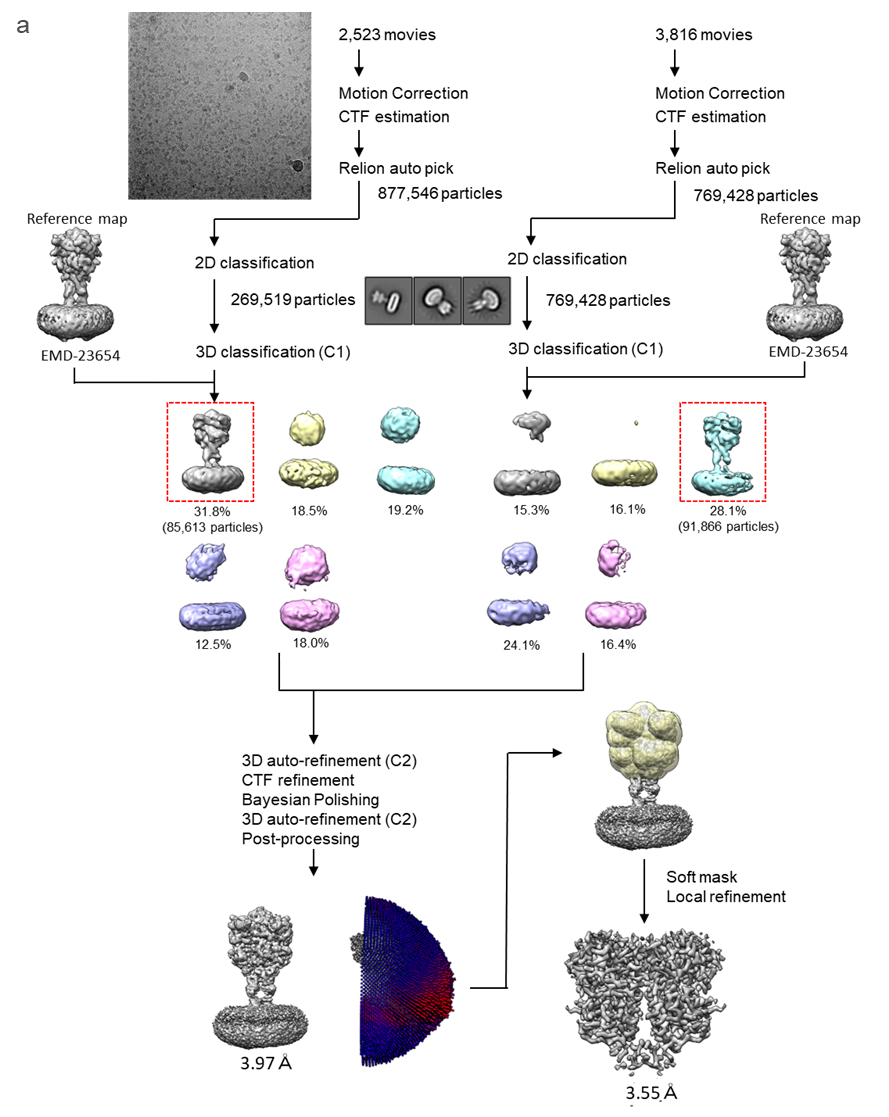
**

**
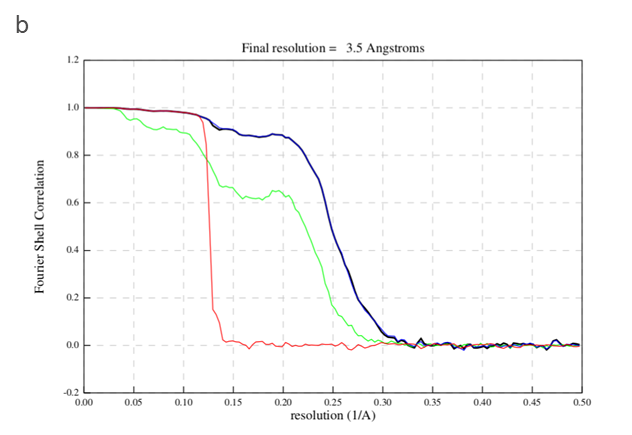
**

**
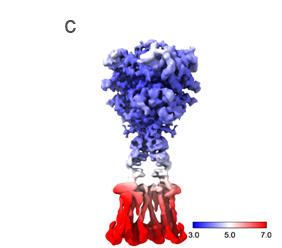

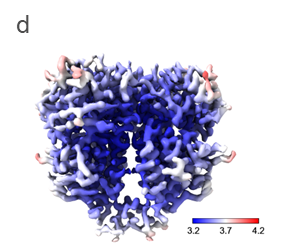
**

**Supplementary Figure 1. Cryo-EM analysis flow chart of cryo-EM data processing for the CaSR/γ-EVG complex.** (**a**) Processing workflow for the CaSR/γ-EVG structure determination by single-particle analysis. (**b**) Gold-standard FSC curves for CaSR/γ-EVG are displayed after applying no mask (green), a mask (blue), or a phase-randomized mask (red). The corrected FSC curve is shown as a black line. All the images in this figure were created in UCSF Chimera. (**c**) Local resolution of the overall map. Color bars show the resolution (Å). (**d**) Local resolution of the VFT domain after local refinement. Cryo-EM: cryo-electron microscopy; CaSR: calcium-sensing receptor; γ-EVG: γ-glutamyl-valyl-glycine; FSC: Fourier shell correlation; VFT: Venus flytrap; CTF: contrast transfer function.

**
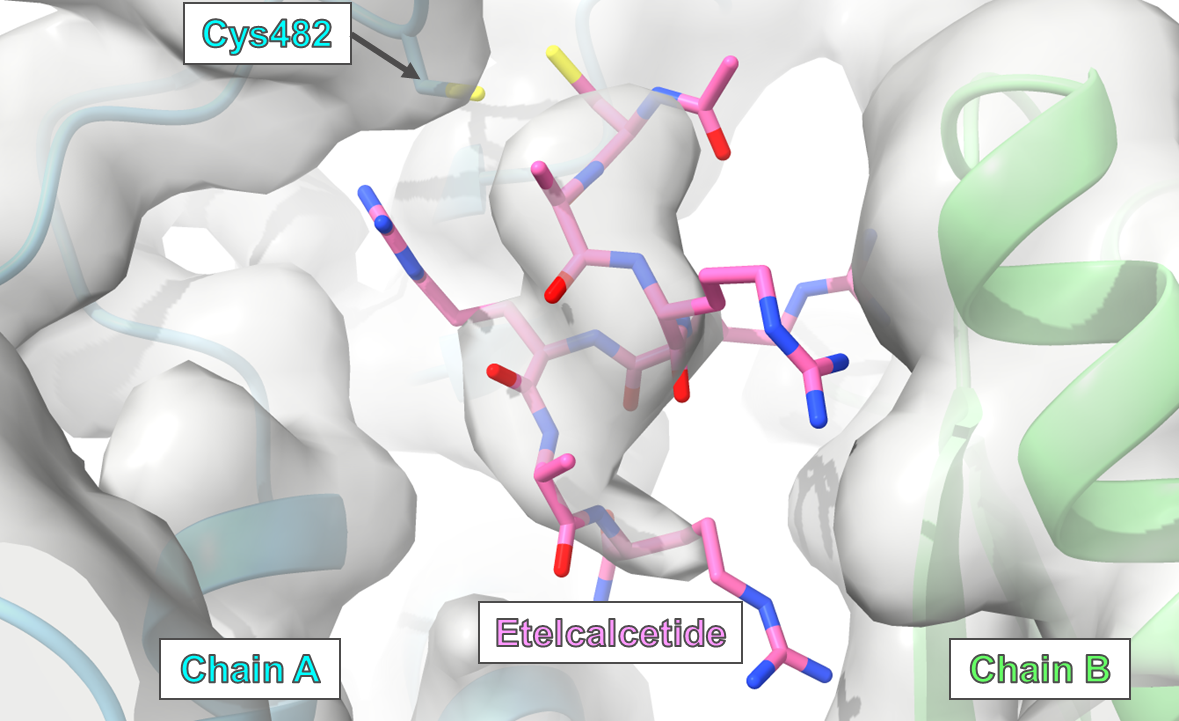
**

**Supplementary Figure 2. Density map around residue Cys482 with a Gaussian filter** The density map with a 1.5 Å Gaussian filter applied is shown in gray. Chains A and B of CaSR are shown as a cartoon models (cyan and green, respectively). A stick model structure of etelcalcetide (pink, PDB: 7M3G) is shown with both CaSR chains superimposed. CaSR: calcium-sensing receptor.

**
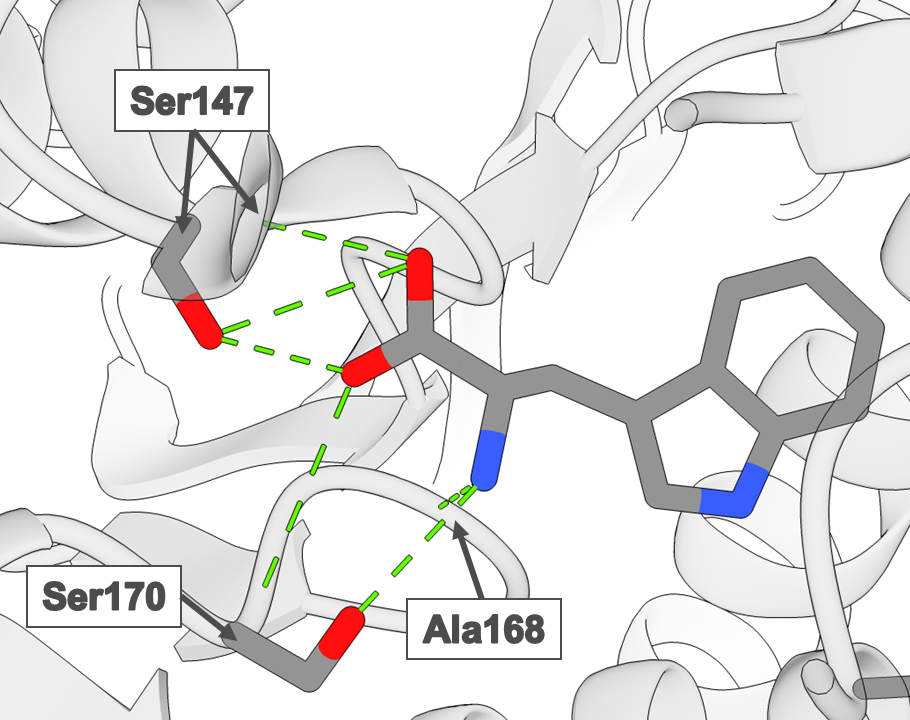
**

**Supplementary Figure 3. Hydrogen bonds between CaSR and L-Trp.** Hydrogen bonds are shown as green dotted lines in the CaSR/L-Trp structure (PDB: 7M3G) using ChimeraX^36^. CaSR is shown as a cartoon model (white) and both L-Trp and the side chains of residues that interact with L-Trp are shown as stick models (gray). CaSR: calcium-sensing receptor.


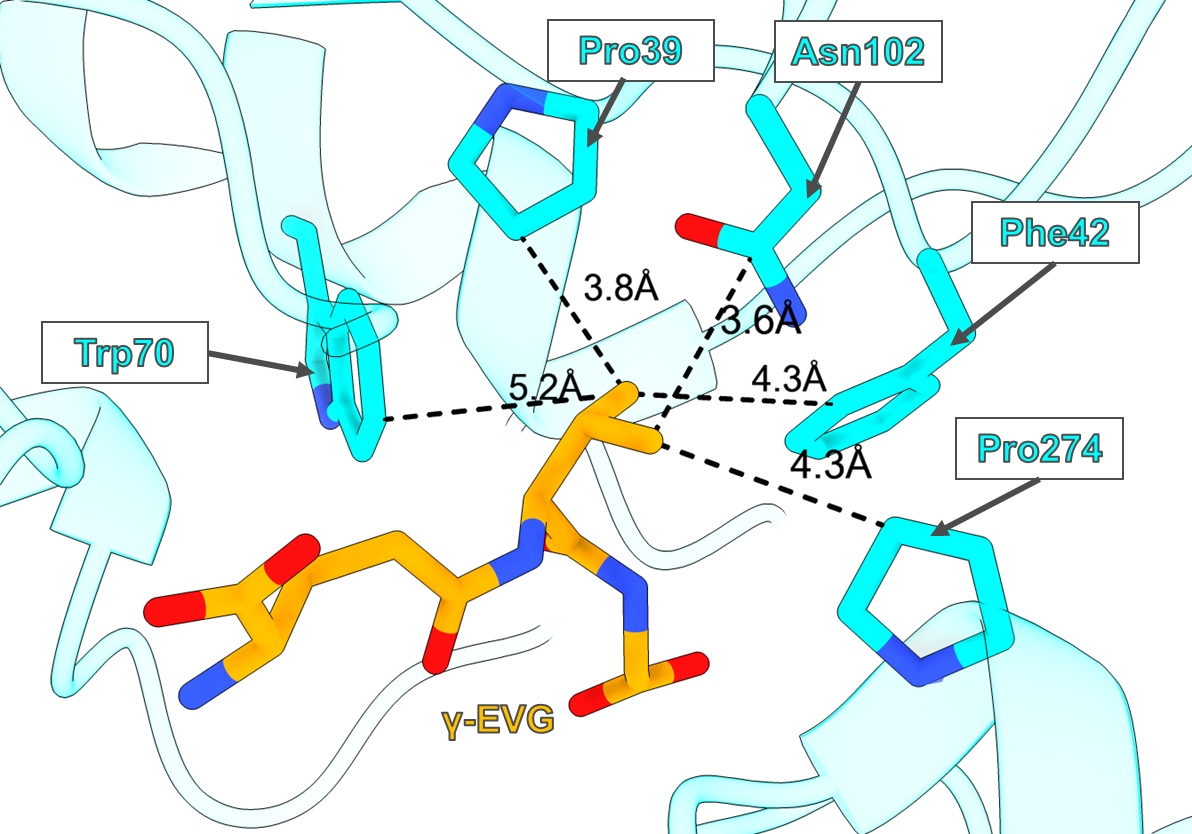


**Supplementary Figure 4. Residues of CaSR around the second position of γ-EVG.**

Dotted lines show distances between CaSR and the valine residue of γ-EVG. CaSR is shown as a cartoon model (cyan) and the side chains of residues that interact with γ-EVG are shown as stick models. γ-EVG is also shown as a stick model (orange). CaSR: calcium-sensing receptor; γ-EVG: γ-glutamyl-valyl-glycine.


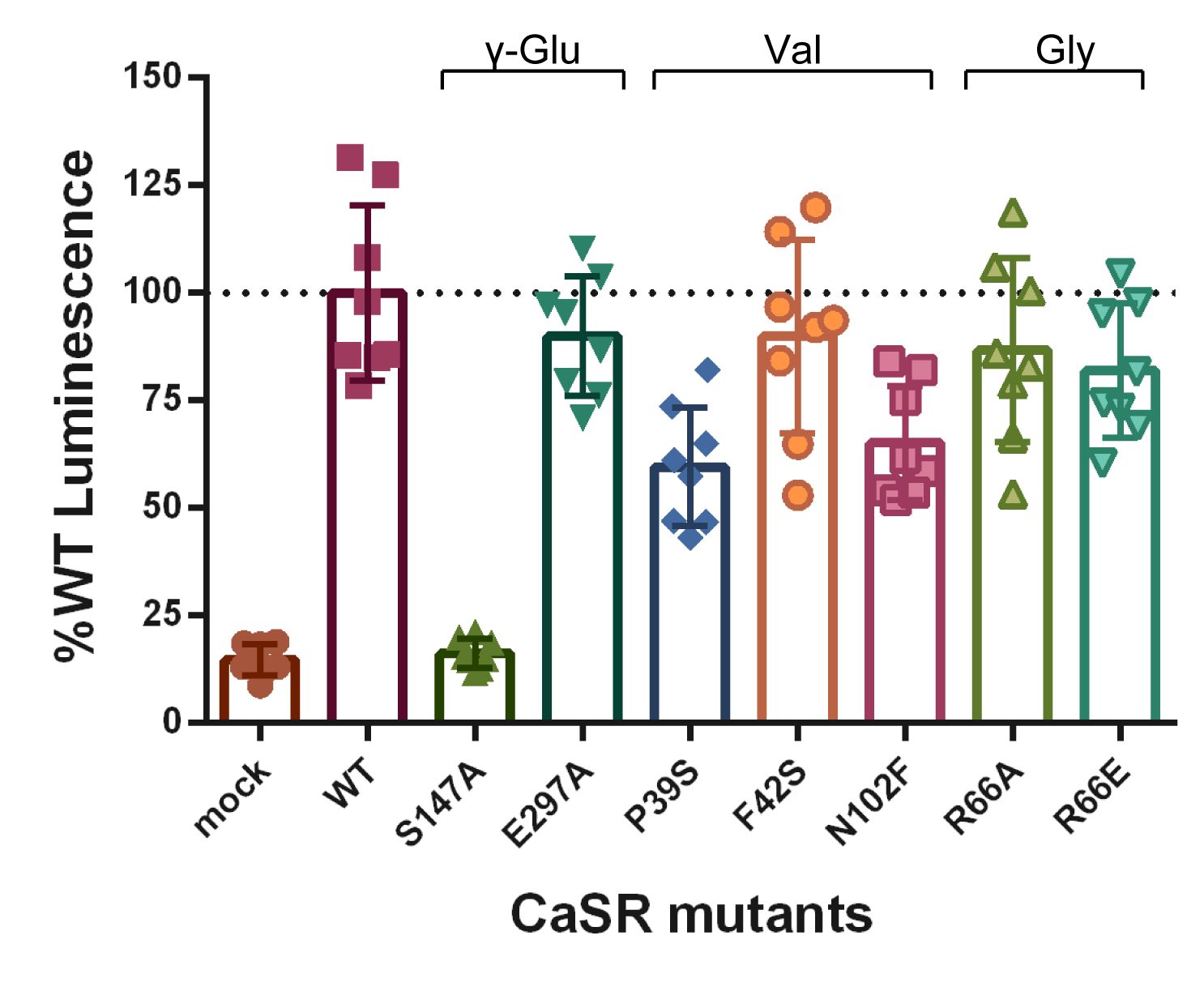


**Supplementary Figure 5. Measurement of the cell-surface expression level of CaSR mutants.** PEAK^rapid^ cells were used to express each construct and the expression levels were measured by ELISA. Data are mean ± s.e.m. from eight independent experiments (*n* = 8). CaSR: calcium-sensing receptor; WT: wild type;

**Supplementary Figure 6. Dose response curves of key residue mutations in ligand binding pockets and receptor activation for L-Tyr in CaSR in a calcium mobilization assay.**

(**a–c**) Dose–response curves of key residue mutations in CaSR in response to stimulation with L-tyrosine in a calcium mobilization assay. Data are mean ± s.e.m. from three independent experiments (*n* = 3), CaSR: calcium-sensing receptor; WT: wild type.

**Table S1 EC_50_ values for CaSR ligands of wild-type CaSR and mutants**

| EC_50_ | WT | E297A | P39S | F42S | N102F | R66A | R66E |
| --- | --- | --- | --- | --- | --- | --- | --- |
| CaCl_2_ (mM) | 0.19 ± 0.4 | 6.58 ± 0.3 | 7.52 ± 0.23 | 6.60 ± 0.3 | 2.75 ± 0.21 | 3.87 ± 0.5 | 3.47 ± 0.4 |
| L-Tyr  (mM) | 3.90 ± 1.1 | NA | NA | NA | 2.16 ± 0.4 | NA | NA |
| γ-EV  (μM) | 10.71 ± 0.1 | NA | NA | NA | 4.48 ± 0.2 | NA | NA |
| γ-EVG  (μM) | 0.18 ± 0.1 | NA | NA | NA | 6.52 ± 0.1 | 18.94 ± 0.5 | NA |

EC_50_ values were calculated from the concentration–response curves (Fig. 4). Data are shown as mean ± s.e.m. from three independent experiments (*n* = 3). NA: not analyzed; CaSR: calcium-sensing receptor; γ-EV: γ-glutamyl-valine; γ-EVG: γ-glutamyl-valyl-glycine; WT: wild type.

**Table S2** ***E*_max_ for CaSR ligands of CaSR and mutants**

| E_max_ | WT | E297A | P39S | F42S | N102F | R66A | R66E |
| --- | --- | --- | --- | --- | --- | --- | --- |
| CaCl_2_ (mM) | 100 | 86.0 | 74.5 | 87.0 | 87.8 | 107.2 | 105.0 |
| L-Tyr  (mM) | 100 | NA | NA | NA | 131.9 | NA | NA |
| γ-EV  (μM) | 100 | NA | NA | NA | 194.8 | NA | NA |
| γ-EVG  (μM) | 100 | NA | NA | NA | 95.7 | 53.6 | NA |

*E*_max_ values were calculated from the concentration–response curves (Fig. 4). Data are shown as mean ± s.e.m. from three independent experiments (*n* = 3). *E*_max_: maximum response; NA, not analyzed; CaSR: calcium-sensing receptor; γ-EV: γ-glutamyl-valine; γ-EVG: γ-glutamyl-valyl-glycine.
